# Antisense oligonucleotide therapies for monogenic disorders

**DOI:** 10.1515/medgen-2025-2025

**Published:** 2025-07-17

**Authors:** Ilona Krey-Grauert, Irene Ferro, Matias Wagner

**Affiliations:** University of Leipzig Medical Center Leipzig Institute of Human Genetics Philipp-Rosenthal-Str. 55 04103 Leipzig Germany; Technical University of Munich Institute of Human Genetics Trogerstr. 32 81675 Munich Germany; Technical University of Munich Institute of Human Genetics Trogerstr. 32 81675 München Germany

**Keywords:** Antisense oligonucleotides, RNA therapeutics, precision medicine, gene therapy

## Abstract

Antisense oligonucleotides (ASOs) are a promising therapeutic modality for monogenic disorders, offering precise RNA-targeting strategies to modulate gene expression. Despite challenges in delivery, toxicity, and off-target effects, ASO therapies have advanced rapidly, with several approved treatments and numerous candidates in clinical development. Their application spans neurogenetic, metabolic, and oncologic disorders, also with emerging n-of-1 approaches for ultra-rare conditions. This review describes the different mechanism of how ASOs work depending on their chemistry and discusses the considerations of which patients could be amendable for treatment highlighting the role of human genetics for decision making.

## Introduction

Already in 1978, Stephenson and Zamecnik pioneered oligonucleotide-based therapies by designing a short oligonucleotide capable of targeting parts of the Rous sarcoma virus transcript thereby reducing viral replication.^1^ Their discovery not only revealed the unexplored potential of antisense therapies but also generated the need to improve every aspect of this technology with the aim of using Antisense oligonucleotides (ASOs) as a corrective tool for monogenic diseases.

ASOs are small non-coding nucleic acids designed to bind target RNA specifically, thereby modulating gene expression at the RNA level. Even though the number of approved ASOs by the European Medicines Agency (EMA) is still limited, the number of ASOs that have entered clinical trials is growing constantly.^2^

Within the field of rare diseases, ASOs represent a rapidly advancing class of drugs that have opened new avenues for the treatment of a wide variety of genetic disorders, especially monogenic diseases. The design and application of ASOs depends on understanding the underlying pathomechanism of the respective disorder determining ASO properties.

In addition to treatment of rare monogenic disorders, ASOs are being explored for their potential in oncology. By targeting the mRNA of oncogenes or other critical proteins involved in cancer cell survival, ASOs can inhibit tumor growth. This strategy is particularly promising for cancers with somatic mutations that are difficult to target with conventional small molecules or monoclonal antibodies. ASOs targeting mutant forms of the *KRAS* gene, for instance, are currently under investigation in clinical trials.^3^

The versatility of ASOs, combined with their potential for precision medicine, has sparked intense interest in both academic research and the pharmaceutical industry. Ongoing advancements in ASO design, delivery technologies, and clinical trials continue to expand the range of diseases that could potentially benefit from this innovative approach to therapy. ASOs and their ability to specifically target RNA and modulate gene expression are still in their infancy for the treatment of a monogenic disorders. While challenges remain in terms of delivery, toxicity and off-target effects, the success of ASOs in clinical applications has already demonstrated their potential to change the landscape of therapeutic development.^2^

This review aims to provide an overview of ASO application for monogenic diseases covering basic mechanisms of action, eligible conditions and patients, chemical properties and ASOs with FDA approval as well as currently being investigated in clinical trials. Moreover, it aims to discuss the current gap between approved therapies and n-of-1 approaches as well as the caveats in therapeutic application.

## Basic mechanism of ASOs

ASOs interact with RNA through various mechanisms, depending on their chemical modifications and sequence complementarity to the target transcript. The fundamental principle underlying ASO function is sequence-specific RNA binding, which facilitates targeted modulation of RNA processing. This specificity allows for relatively straightforward ASO design and application in *in vitro* settings.

One key mechanism by which ASOs exert their effects is steric blockade, where the ASO binds to a target RNA sequence and physically prevents the interaction of RNA-binding proteins. This approach can be employed to inhibit the binding of translation initiation factors, thereby reducing protein synthesis. However, it is more commonly used to modulate pre-mRNA splicing by interfering with the binding of splicing factors. This strategy is exemplified by splice-switching ASOs (SSOs), such as *nusinersen*, which is used to treat spinal muscular atrophy (SMA). SMA (OMIM: #253300) is caused by biallelic variants in *SMN1* resulting in a deficiency of survival motor neuron (SMN) protein. However, the paralogous gene *SMN2* predominantly produces a truncated, nonfunctional SMN protein due to alternative splicing that excludes exon 7. *Nusinersen* binds to a regulatory sequence in intron 7 of *SMN2* pre-mRNA, preventing the association of splicing repressors. This promotes exon 7 inclusion in the mature mRNA, enabling the production of full-length SMN protein, thereby restoring motor neuron function and slowing disease progression.^5^

A similar approach was used in an n-of-1 treatment for a child with Batten disease (CLN7, OMIM #610951), a form of neuronal ceroid lipofuscinosis.^6^ The causative variant was a deep intronic pathogenic variant in the *MFSD8* gene, leading to the inclusion of a pseudo exon and subsequent loss of function. The personalized ASO *milasen* was designed to bind the cryptic exon recognition sequence within *MFSD8* pre-mRNA. By sterically blocking the spliceosome machinery, *milasen* prevented pseudo exon inclusion, restoring proper splicing of *MFSD8* mRNA. This correction enhanced MFSD8 protein production, improving lysosomal function and potentially mitigating neurodegeneration.

Targeting aberrant splicing events, however, is difficult when pathogenic variants disrupt the canonical splice site as ASOs targeting these regions in the pre-mRNA prevent binding of splicing factors usually resulting in exon skipping and likewise a loss of function. Hence, deep intronic or exonic splice variants are better targets for splice correction employing ASOs.^7^

While splice-switching ASOs are primarily used to correct aberrant splicing and restore physiological gene function, gapmer ASOs utilize a different mechanism of action. Gapmers are single-stranded hybrid oligonucleotides composed of a central DNA “gap” flanked by chemically modified RNA-like nucleotides. Upon binding to a complementary RNA sequence, the resulting DNA-RNA duplex is recognized by RNase H, which specifically degrades the target RNA. This mechanism makes gapmer ASOs particularly useful for reducing the expression of pathogenic transcripts.^2^

Gapmer ASOs can be designed for either non-selective or allele-preferential RNA degradation. In disorders caused by gain-of-function mutations, such as those with dominant-negative effects, non-selective knockdown of both alleles may be detrimental.^2^ Instead, allele-preferential ASOs targeting a causative variant or exploiting single-nucleotide polymorphisms (SNPs) to distinguish between mutant and wild-type alleles are preferred. A perfectly complementary ASO will bind the mutant transcript with high affinity, whereas a single nucleotide mismatch with the wild-type allele reduces binding efficiency, thereby conferring selectivity. This strategy is particularly relevant when a single recurrent mutation is responsible for disease pathogenesis, as seen in TTR-amyloidosis (OMIM #105210), where mutation-specific ASOs are preferred over non-selective knockdown approaches.^8^

Examples of non-selective gapmer ASOs include *elsunersen*, currently in phase 3 clinical trials for *SCN2A* gain-of-function (GoF) developmental and epileptic encephalopathy (OMIM: #613721)^9^, and *tofersen*, which is approved for the treatment of *SOD1*-associated amyotrophic lateral sclerosis (ALS, OMIM #105400).^10^ Conversely, allele-selective gapmers have been developed for conditions such as *FUS*-ALS (OMIM #608030), where the ASO *jacifusen* selectively targets the pathogenic c.1574C>T (p.Pro525Leu) variant.^11^

However, in the case of loss-of-function mutations, conventional gapmer strategies fail to upregulate wild-type mRNA or restore protein expression. To address this limitation, targeted augmentation of nuclear gene output (TANGO) strategies have been developed to increase mRNA and protein levels through ASO-mediated mechanisms. TANGO comprises three main approaches:

1. Targeting poison exons: This approach utilizes splice-switching ASOs to selectively inhibit naturally occurring, non-productive splicing events, thereby enhancing the production of functional mRNA and protein. A notable example is the application of ASO therapy in a mouse model of *SCN1A*-associated Dravet syndrome, where blocking a non-productive splicing event increased *SCN1A*-mRNA and NaV1.1 protein levels, ultimately reducing seizure frequency. A phase 3 clinical trial employing this strategy is currently ongoing, with preliminary results demonstrating a median seizure reduction exceeding 50 % in Dravet syndrome patients.^12^
2. Targeting long noncoding RNAs (lncRNAs): lncRNAs, which are >200 nucleotides in length, regulate gene expression through diverse mechanisms, including transcription factor recruitment and modulation of epigenetic marks. Additionally, lncRNAs can influence mRNA stability and translation efficiency. For example, *Chaserr*, a lncRNA transcribed near *Chd2*, has been shown to regulate *Chd2* expression. In a mouse model, depletion of *Chaserr* resulted in increased *Chd2* mRNA and protein levels, suggesting that lncRNA-targeting ASOs could be leveraged to restore physiological gene expression.^13^
3. Targeting untranslated region (UTR) elements: ASOs can enhance protein translation by blocking translation-inhibitory elements within mRNA UTRs. These include upstream open reading frames (uORFs), translation-suppressive motifs, and miRNA-binding sites. Additionally, ASOs can be designed to bind and inhibit specific miRNAs that negatively regulate translation, thereby increasing protein output.^14^

**Figure 1:**
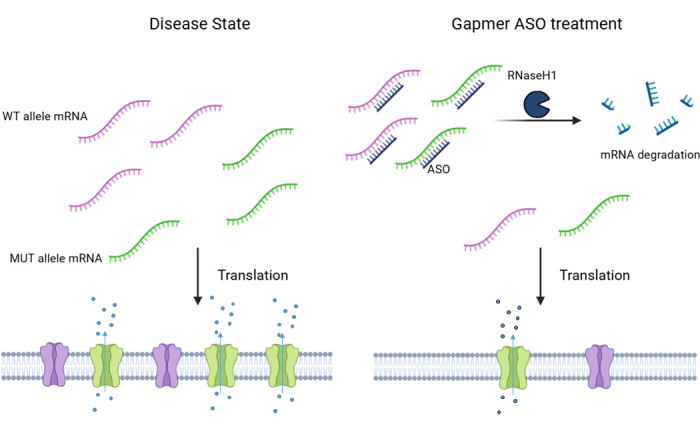
**Mechanism of gapmer ASOs**. Left panel: In the disease state, a heterozygous gain-of-function variant results in the expression of both a WT (wildtype, purple) and a mutant protein, in this example an ion channels with increased permeability (shown in green). Reduction of mRNA levels is induced by treatment with a gapmer ASO. By binding to the target mRNA, the gapmer ASO leads the knockdown of the disease gene through the recruitment and activation of the RNaseH1 enzyme normalizing the electrophysiological properties of the cell. In this case, the gapmer ASO does not target the mutant allele specifically.

Collectively, ASO-based strategies hold therapeutic potential for a broad spectrum of autosomal dominant monogenic disorders, including those caused by heterozygous gain-of-function, dominant-negative, and loss-of-function mutations. ASO treatment for recessive conditions, however, is often more complex and usually employs splice-switching ASOs for correcting pathogenic splicing defects.

## Suitable monogenic conditions and eligible patients for ASO therapies

When applying or designing ASO therapies it is – similarly to enzyme replacement therapies – important to consider which patients with their respective diagnoses might benefit from treatment with repetitive, potentially intrathecal, injections. The bioavailability of antisense oligonucleotides (ASOs) is generally low when administered systemically (intravenously), mainly due to their rapid clearance, degradation by nucleases, and limited cellular uptake.^15^ ASOs primarily accumulate in the liver and kidneys, with uptake mediated by endocytosis. They are eliminated via renal excretion or intracellular degradation. Hence, systemic disorders such as metabolic diseases with multiple directly affected organs are extremely difficult to treat. In contrast, there are key organ systems where ASOs are commonly used^16^:

1. Central Nervous System (CNS): The CNS is a key target for ASOs, especially for neurodegenerative diseases like Huntington’s disease or developmental and epileptic encephalopathies (DEEs). Even though systemic delivery is inefficient due to the blood-brain barrier (BBB), intrathecal administration allows ASOs to bypass the BBB and reach neurons and glial cells both in the brain and the spinal cord directly. The possibility of an intrathecal administration comes with the advantage of a slow ASO clearance from the cerebrospinal fluid.^17^
2. Liver: The liver efficiently takes up ASOs via receptor-mediated endocytosis, making it a suitable target for some metabolic or even infectious diseases (e. g., hypercholesterolemia, hepatitis B). However, metabolic diseases affecting other tissues would benefit only minimally (e. g. mitochondrial disorders).
3. Kidney: ASOs naturally accumulate in renal proximal tubule cells, making them potential candidates for treating kidney-related disorders.
4. Eye: The eye is an attractive target for ASOs in conditions like Leber congenital amaurosis and macular degeneration as direct intravitreal injections achieve high local concentration minimizing systemic exposure to reduce side effects.

**Figure 2:**
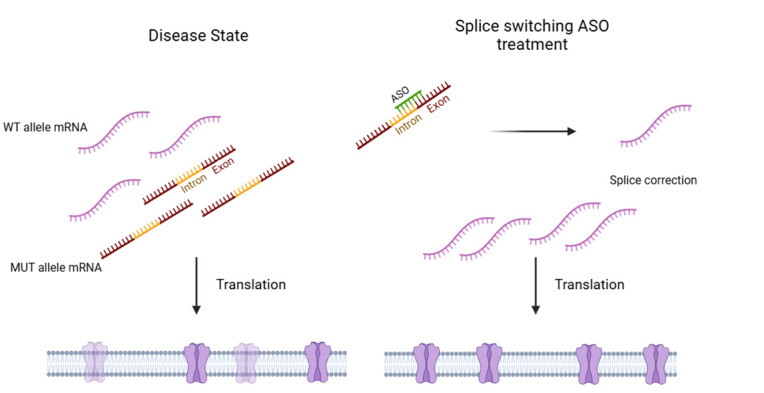
**Mechanism of Splice Switching ASOs.** Left panel: In the disease state, a heterozygous pathogenic variants causes a splice defect, in this case, an intron retention resulting in a mutant pre-mRNA (MUT allele mRNA). Mis-splicing results in reduced protein translation displayed as transparent ion channels. Right panel: The splice switching ASO (SSO) targets the mutant pre-mRNA correcting splicing by skipping of the kryptic exon resulting in physiological protein levels.

Additionally, it is important to consider the course of a disease potentially suitable for ASO treatment.

Degenerative and progressive diseases are especially good candidates for ASO therapy because they often result from the gradual accumulation of toxic proteins or the depletion of essential ones resulting in progressive cell death and organ failure. In these cases, an ASO that either enhances the production of a deficient protein or silences the respective RNA can significantly alter disease progression. A prime example is Huntington’s disease, where ASOs might reduce the levels of mutant huntingtin RNA slowing neurodegeneration (no FDA approved therapy, yet).^18^

In contrast, diseases where genetic variation leads to developmental malformations–such as congenital structural defects–are less amenable to ASO therapy. If a mutation disrupts early embryonic development, leading to irreversible structural abnormalities, modifying RNA expression later in life may have little therapeutic benefit.

Ultimately, ASO therapies are particularly well-suited for monogenic disorders where disease mechanisms involve aberrant splicing (treatment with splice switching ASOs), toxic RNA accumulation, gain-of-function or dominant negative mutations (treatment with gapmer ASOs, potentially even allele selective gapmer ASOs). It is more difficult but still feasible to treat conditions caused by haploinsufficiency as ASOs will have to upregulate expression of the wildtype allele which can be achieved by targeted augmentation of nuclear gene output (TANGO, see STK-001 targeting a poison exon in *SCN1A*). However, monogenic disorders where variants result in a biallelic loss-of-function are currently not suitable for ASO treatment (only if splice variants can be targeted with splice-switching ASOs). Only in very few cases, where ASOs can introduce or improve a compensatory mechanism, recessive conditions are suitable such as in *SMN1*-related spinal muscular atrophy where ASOs target indirectly the disease associated gene.

## Structure of ASOs

Nucleic acids are susceptible to rapid degradation by endo- and exonucleases within a few seconds both *in vitro* and *in vivo*. For this reason, the last two decades of research have focused not only on the identification of monogenic diseases suitable for treatment with ASOs, but also on the identification of chemical modifications that enable ASOs to acquire drug-like properties: greater resistance to degradation, binding affinity to the substrate, a safe toxicological profile, and a pharmacokinetics favourable for clinical application.

Numerous modifications have been identified to develop increasingly potent nucleotide analogues. These modifications can be divided into three major groups based on the part of the nucleotide structure that is modified: internucleotide linkages, sugar moieties, and nucleobase modifications.^2^ The following modifications have been most successful in leading to FDA approval:

### Internucleotide linkages modifications:

To address the challenge of vulnerability to enzymatic digestion, the first aspect of antisense oligonucleotide chemistry that has been modified is the phosphodiester bond connecting nucleotides, also known as the backbone. Here, the non-bridging oxygen of the phosphate group can be replaced by a sulphur atom to form a phosphorothioate (PS) linkage. ASOs designed with a PS-backbone show greater resistance to nucleases as well as higher binding affinity to plasma and intracellular proteins, which improves ASOs’ pharmacodynamic profile. Furthermore, the higher lipophilicity conferred by PS linkages promotes uptake by cells and tissues. However, the introduction of a PS backbone has its downsides as well. The presence of the PS backbone results, on one hand, in a decrease in the binding affinity for the target RNA and, on the other hand, in a higher affinity for immune receptors, which can lead to an undesired pro-inflammatory response.^19^ Nonetheless, phosphorothioate bonds are currently the most commonly used modification in the design of ASOs, in fact, all gapmers FDA-approved or in clinical trials so far include a full-PS-backbone or a mix of PO and PS linkages.

Phosphorodiamidate morpholino oligonucleotides (PMOs) is the most common chemical modification for splice-switching antisense oligonucleotides (SSOs) that are systemically administered. This chemical modification changes both the phosphate bond and the sugar ring constituting the nucleotide structure: a morpholine ring substitutes the furanose ring and a neutral phosphorodiamidate backbone replaces the PO linkage. The result of such chemical modification is a full protection from nuclease degradation and a safer profile compared to a PS backbone due to low binding affinity to plasma proteins. To date, several ASOs containing PMOs are FDA-approved for the treatment of Duchenne Muscular Dystrophy (DMD).

### Sugar moieties modifications:

Sugar moieties have been, together with the backbone, the most studied and heavily modified structures to make ASOs suitable for clinical applications. Generally, these analogues involve the modification of the sugar in 2‘ position and are aimed to confer a stronger binding affinity for the target mRNA and nuclease resistance. Thanks to their high affinity for RNA and their low immunogenicity, 2′-O-methyl (2′-OMe) and 2′-O-methoxyethyl (2′-MOE) were the first sugar modifications to be introduced are still the preferred choice for splicing-modulating ASO approach.^20,^ ^21^ Another promising modification for SSOs is 2′-fluoro (2′-F), which demonstrated exceptionally exon skipping capability.^22^

More recent modifications are Locked Nucleic Acids (LNAs), in which the 2′-oxygen and the 4′-carbon of the sugar ring are connected by a methylene bridge. This chemical structure constrains the sugar ring into a “locked” conformation that increases the stability of the nucleic acid strand, enhances binding affinity to complementary RNA or DNA, and improves resistance to enzymatic degradation. LNA modification is so far the most promising to improve gapmer ASO potency. Despite the advantages, LNA-containing gapmers show an increased toxic *in vivo* profile, which has so far prevented its therapeutic application.

### Nucleobase modifications:

The only modification currently utilized for the nucleobase is the methylation of cytosine at the 5’ position (5‘-mC). Widely used both in gapmers and splice-switching ASOs, this modification drastically improves the toxicological profile by reducing the immune response against unmethylated CpG regions.

### Approved and upcoming ASO therapies

Table 1 provides an overview of approved ASOs (FDA and EMA). Additionally, certain ASOs in the field of developmental and epileptic encephalopathies (DEEs) have entered development with examples being shown in Table 2. Clinical studies on DEEs will demonstrate if ASO treatment will primarily reduce seizure burden in these conditions and constitute an advanced symptomatic therapy or improve cognition, behavioral symptoms, interaction and motor skills as well and therefore represent a true disease modifying therapy. Preliminary results from phase I studies appear promising (e. g. press release Stoke therapeutics, March 25, 2024). The results of phase 3 studies on cognition and behavior in DEEs will be essential to evaluate the use of ASOs for patients with (non-syndromic) intellectual disability that are mostly poorly understood on a connectome level and where a causative genetic alteration potentially results in abnormal brain development that could not or only minimally be corrected by postnatal interventions. 

**Table 1:** Overview of Approved ASOs (FDA and EMA).

Name	Target	Indication	Organ (ROA)	Chemistry	FDA approval	EMA approval	Company
Mipomersen (Kynamro)	*APOB*	Homozygous familial hypercholesterolemia	Liver (SQ)	gapmer ASO	2013	refused	Ionis Genzyme Kastle
Eteplirsen (Exondys 51)	*DMD* exon 51	Duchenne muscular dystrophy	Skeletal muscle (IV)	steric block ASO	2016	refused	Sarepta
Nusinersen (Spinraza)	*SMN2* exon 7	Spinal muscular atrophy	Spinal cord (IT)	steric block ASO	2016	2017	Ionis Biogen
Inotersen (Tegsedi)	*TTR*	Hereditary transthyretin amyloidosis	Liver (SQ)	gapmer ASO	2018	2018	Ionis Akcea
Golodirsen (Vyondys 53)	*DMD* exon 53	Duchenne muscular dystrophy	Skeletal muscle (IV)	steric block ASO	2019	no	Sarepta
Volanesorsen (Waylivra)	*APOC3*	Familial chylomicronemia syndrome	Liver (SQ)	gapmer ASO	2019	2019	Akcea
Casimersen (Amondys 45)	*DMD exon 45*	Duchenne muscular dystrophy	Skeletal muscle (IV)	steric block ASO	2021	no	Sarepta
Tofersen (Qalsody)	*SOD1*	Amyotrophic lateral sclerosis	CNS (IT)	gapmer ASO	2023	2024	Biogen
Eplontersen (Wainzua)	*TTR*	Hereditary transthyretin amyloidosis	Liver(SQ)	gapmer ASO	2023	pending	AstraZeneca

Overview of approved ASOs. *ROA: route of administration, IT: intrathecal, SQ: subcutaneous, NS: nervous system, IV: intravenous, CNS: central nervous system, ASO: antisense oligonucleotide*

## Approved therapies vs. n-of-1 approaches

ASO therapies have gained significant traction, with multiple treatments receiving regulatory approval for conditions where they offer a clear therapeutic advantage. Pharmaceutical companies primarily focus on developing ASOs for prevalent genetic diseases that affect larger patient populations and are relatively accessible for treatment. By targeting diseases with well-defined genetic mechanisms and established clinical endpoints, pharmaceutical companies can justify the substantial investment required for large-scale clinical trials and regulatory approval.

In contrast, n-of-1 ASO therapies have emerged as a highly personalized approach, aiming to treat ultra-rare or even patient-specific mutations. These therapies, such as milasen–developed for a single patient with Batten disease^6^–demonstrate the flexibility of ASO drug development, as they can be rapidly designed and tested on an individual basis. However, the costs associated with developing these personalized therapies are extraordinarily high, often exceeding several million dollars per patient.^23^ This raises concerns about their long-term sustainability, as current regulatory frameworks, manufacturing processes, and reimbursement models are not designed to support treatments that benefit only one or very few individuals.

Bridging the gap between widely approved ASO therapies and n-of-1 treatments will require innovative approaches to drug development, regulation, and funding. One potential solution is the creation of platform-based approval pathways, where regulatory agencies could approve ASO scaffolds with predefined chemical backbones and nucleotide modifications, allowing for faster adaptation to new targets without requiring full clinical trials for each individual case. Another promising avenue is the development of collaborative funding models, where public health systems, private insurers, and philanthropic organizations share the financial burden of n-of-1 treatments, ensuring that life-saving therapies remain accessible without creating unsustainable economic pressure.

Ultimately, the future of ASO therapy will depend on balancing the pharmaceutical industry’s focus on prevalent conditions with a growing claim for individualized treatments. By refining regulatory pathways, improving cost efficiency, and fostering collaborative funding strategies, it may be possible to make personalized ASO therapies a viable option for more patients in need.

**Table 2:** Selected oligonucleotide therapeutics for the treatment of neurogenetic disorders that have entered development. ROA: route of administration, IT: intrathecal, CNS: central nervous system.

Name	Target	Indication	Organ (ROA)	Company	Clinical trial stage
STK-001 (Zorevunersen)	*SCN1A*	Dravet syndrome	CNS (IT)	Stoke Therapeutics	Phase III
PRAX-222 (Elsunersen)	*SCN2A*	SCN2A-DEE	CNS (IT)	Praxis Precision Medicines	Phase III
GTX-102	*UBE3A*	Angelman syndrome	CNS (IT)	Ultragenyx	Phase III

## Side effects and toxicity

Two types of AOS associated toxicity can be distinguished: sequence-dependent and sequence-independent. The first type is mostly due to ASOs binding to off-target sequences such as intronic regions^24^ and long pre-mRNA transcripts.^25^ Sequence-independent toxicity occurs when PS-ASOs interact with intracellular proteins, especially by forming disulphide bonds, therefore leading to protein mislocalisation, nucleolar stress and apoptosis.^26^ Protein-binding derived toxicology can be prevented by accurate design of ASOs to avoid sequence motifs and modification patterns that are likely to bind with off-target proteins.

Additionally, it is important to highlight that all chemical modifications significantly influence the toxicological profile of ASOs. While 2‘-OMe and 2’-MOE modifications have contributed to the therapeutic success of several splice-switching and gapmer ASOs, LNAs have not progressed past clinical trials due to increased toxicity.^27^ Generally, heavily modified ASOs tend to be more toxic than less modified ASOs due to the increased binding affinity for plasma proteins that these modifications provide complicating drug development endeavours.

Different ASOs adverse effects can be observed based on routes of administration. PS-ASOs subcutaneously injected can cause injection site reactions (IRS), which are typically mild and can be avoided by lowering the dosage or by the presence of specific modifications.^28^ Hepatotoxicity and nephrotoxicity have been reported in the context of ASO treatments, especially for LNAs that show chemistry-, sequence- and design-dependent hepatotoxicity.^29^ Therefore, ASO therapies have found wide application in the context of brain diseases due to the advantage of the blood-brain barrier (BBB), which restricts the systemic distribution of ASO and minimizes systemic side effects. However, intraventricular injections in mice can result in acute neurotoxicity. Upon injection, ASOs drastically reduce intracellular Ca2+ concentration in neurons in an ASO dose-, sequence-, and modification-dependent manner.^30^ By chelating divalent cations, ASOs create a dysregulation of calcium homeostasis leading to an impaired neuronal activity and, eventually, neuronal death.

## Role of human genetics in ASO precision therapies

Human geneticists are likely to play a central role in the clinical application of antisense oligonucleotide (ASO) therapies by unravelling the precise molecular aetiology of genetic disorders. Through comprehensive variant interpretation and functional classification–such as distinguishing between gain-of-function, loss-of-function, or splicing defects–geneticists provide critical insights that inform the suitability and design of ASO strategies. In clinical settings, they are uniquely positioned to evaluate whether both the disease mechanism and the individual patient meet the molecular and clinical criteria for ASO-based intervention, making them indispensable in selecting eligible candidates and guiding precision therapy development.

Looking forward, the contribution of human genetics will likely extend beyond diagnostics. Geneticists are integral to patient stratification in clinical trials, variant prioritization, and the identification of novel RNA targets. They contribute to natural history studies by genotype phenotype correlation that inform therapeutic timing and trial design. Moreover, genetic reasons might be critical in understanding variability in treatment response, guiding personalized adjustments, and addressing ethical considerations in highly individualized approaches such as n-of-1 therapies. As ASO therapies evolve, human geneticists will be instrumental in shaping diagnostic pathways, treatment frameworks, and access policies, solidifying their role as key enablers of precision medicine.

## Outlook

When looking at the number of EMA approved ASOs it becomes clear that the era of ASO therapies is still in its infancy. Nevertheless, several clinical studies have failed in recent years such as tominersen for the treatment of Huntington disease or rugonersen for the treatment of Angelman syndrome. In these cases, the risk-befit ratio turned out to be unfavorable leading companies to discontinue clinical studies. Hence, compounds need to be improved in their potency which will improve clinical benefit and reduce side effects at the same time. Recent advances in the design and chemistry of ASOs have improved their stability, specificity, and pharmacokinetics, leading to more effective and safer therapies.

While the potential for ASOs as therapeutic agents is immense, several challenges must be addressed for their widespread adoption and success in clinical practice. One of the most significant challenges is the effective delivery of these molecules to the target tissues. ASOs need to be delivered efficiently across cellular membranes and into the cytoplasm of the target cell type in the target organ, where they can bind to their mRNA targets. Especially the technologies of LNPs and conjugating chemistry will significantly improve not only ASO delivery but also toxicity given the better biodistribution of ASOs.

Additionally, long-term effects of ASO therapies are still being evaluated, especially for monogenetic disorders that require prolonged treatment. Ensuring that ASOs maintain their efficacy over time without inducing resistance or unwanted side effects is crucial for their continued success in clinical settings.

Despite all challenges, the future of ASOs in treatment of monogenic diseases is promising. Ongoing research and clinical trials are likely to expand the range of diseases that can be treated with ASO-based therapies, and advancements in delivery methods and molecular design will improve their effectiveness and safety.

With the advances of ASO therapies, especially in an n-of-1 setting, the role of human geneticist becomes increasingly important as experts on the course of a disease and genetic basis as well as the pathomechanism to evaluate the applicability of an ASO therapy. As the field of rare diseases is moving from sole reading nucleic acids for diagnosing patients to an increasing focus on therapeutic options it is important to consider the possibility of writing nucleic acids (ASO therapies) to ameliorate symptoms.
